# A School Story, Not a Student Story: The Dyslexic Diagnosis Paradigm in Children’s and Young Adult Literature

**DOI:** 10.1007/s10583-023-09529-9

**Published:** 2023-05-29

**Authors:** Elizabeth Leung

**Affiliations:** The Centre for Research in Children’s Literature at Cambridge, Faculty of Education, University of Cambridge, 184 Hills Road, Cambridge, CB2 8PQ UK

**Keywords:** Dyslexia, Disability studies, School story, Diagnosis

## Abstract

Representations of dyslexia have a history of educational and literary scholarship primarily concerned with how dynamic characters with learning disabilities are and if they are positively portrayed. This article uses narrative theory to analyze how diagnosis operates on a structural level to create what I call the dyslexic diagnosis paradigm. Examining school stories featuring characters with dyslexia published between 2007 and 2020, I demonstrate how this paradigm functions through a structural closure of struggle, diagnosis and accommodations, and a psychological closure consisting of shame, declaration, and acceptance within these novels. Variations or polytypes of this narrative are also common within this corpus which maintain the psychological closure of shame, declaration, and acceptance present within the prototypical narrative. While some disability counternarratives or dyslexic persistence narratives nuance the school story, the dyslexic diagnosis paradigm ultimately remains prevalent and upholds the medical model of disability within the educational system, promoting the flawed status quo of disability rather than asking readers to question the validity of the systems which enforce them.

## Introduction

When considering characters with dyslexia in contemporary children’s school stories, it is perhaps unextraordinary to say that most dyslexic narratives are diagnosis narratives. Dyslexia is “a specific learning disability […] characterized by difficulties with accurate and/or fluent word recognition and by poor spelling and decoding abilities” (IDA, 2002). As it is neurobiological in origin (IDA, 2002), the dyslexic mind is considered neurodivergent from birth. However, dyslexia is not *evident* at birth; the understanding of one’s dyslexia develops as we encounter the written word and are Othered by ‘Lexism’—the “normative practices and assumptions of literacy” (Collinson, 2012, p. 63)—placed in environments which are disabling to us. Contemporary schools, which emphasize the mastery of literacy, are one such environment and a setting that features heavily in children’s and young adult literature.

Many educational systems operate on the medical or deficit model of disability whereby an individual must prove a lack of abilities according to various tests such as the Wechsler Intelligence Scale for Children (WISC) to qualify for accommodations (Grant, 2017, p. 5). As many people with dyslexia first encounter the written word in school, this often-attenuated process of recognition and diagnosis becomes an integral part of the dyslexic experience. While various scholars have analysed representations of dyslexia in children’s and young adult fiction (Prater, 2003; Altieri, 2008; Leininger et al., 2010) and some celebrate the positive and dynamic representations of characters with dyslexia (Dyches et al., 2001; Jozwik and Rice, 2020), none have focused on how diagnosis remains overly represented or how these representations actively reinforce the system which disables them. I argue that the school story encourages what I call the ‘dyslexic diagnosis paradigm’: a re-occurring narrative structure which features a dyslexic character undergoing diagnosis to receive accommodations so they may succeed within the educational system. This paradigm reinforces the medical model of disability—the *necessity* of a proper diagnosis—celebrating the current system rather than questioning its legitimacy; preventing interjections from the social model of disability and thereby upholding the institutional school by the school story.

To clarify, the goal of this article is not to nitpick myths and misconceptions of dyslexia that may be present within this corpus—though instances of nitpicking may occur—but to uncover how the dominant narrative of diagnosis is structured by the conventions of the school story. Although these narratives may not tell us the ‘true’ dyslexic experience, they tell us how this disability is perceived within our contemporary socio-cultural consciousness as children’s literature “reveals a great deal about the prevailing ideologies of a given period” (Pearson and Reynolds, 2012, p. 64). Contemporary school stories encourage the diagnosis paradigm and medical model of disability acting as celebrations of our current—and flawed—education systems rather than questioning how these systems continue to disable those with dyslexia. Following an introduction to the school story and my corpus, this article will introduce the prototypical dyslexic diagnosis narrative and its polytypes. It will also briefly discuss dyslexic persistence narratives: attempted interjects of the social model of disability which nuance the school story, yet ultimately fail to provide lasting change within the educational system for their neurodivergent characters.

## The (Dyslexic) School Story

Despite widespread use, some scholars—particularly in the UK—challenge the term dyslexia; Elliott and Grigorenko question whether it is “synonymous to, or different from, reading disability” (2014, p. x) and find it so problematic that they recommend the term be discontinued (2014, p. 177). Collinson likewise finds the term problematic as it presumes a deficit or medical model of disability (2012, p. 63). Rather, he proposes that “dyslexia can be defined by the existence of Lexism [to be ‘Othered’ and defined against socio-cultural norms of literacy] rather than the more problematic concept of dyslexia” (2012, p. 63). On the sociology and disability activism side of the debate, dyslexia as an identity is embraced within the neurodiversity paradigm wherein neurodivergent minds such as those with dyslexia, autism, AD(H)D, PTSD, mood disorders, etc., are a “natural and valuable form of human diversity” (Walker, 2021, p. 27). While this debate is ongoing within dyslexia studies, I follow other education researchers who continue to use the term dyslexia. Although sometimes referred to as a learning difference, challenge, or difficulty (Jozwik and Rice, 2020), I continue to consider dyslexia a learning disability as people with dyslexia are continually placed in disabling environments.

This Othering by Lexism is heightened within school environments wherein most contemporary realist texts which represent dyslexia are set; contemporary realism and the school story may as well be synonymous when discussing this corpus.^1^ The school story has been present since the beginnings of realist children’s fiction in the eighteenth century. As Reimer argues that early English school stories were made with the “purpose of shaping the minds of child-readers as they prepare[d] to enter the world of school, a world understood to be a place of struggle,” these texts reflected how schools were primarily considered a place to mould children to fit into Imperial England’s capitalist world through constant surveillance (2009, pp. 210, 211). In that sense, these texts are mimetic, reproducing the world of the reader in an “adequate, truthful representation of reality” (Joosen, 2006, para. 1), and the schools act as metaphors for the larger adult world. Legacies of these themes remain in their contemporary counterparts within modern schooling systems, although these modern texts often illustrate microcosms of a corrupted world, rather than an imperialistic paradise (Zipes, 2005, p. 1805).

Despite the school story waning in the early twentieth century (Pesold, 2017, p. 2), children’s literature saw a revival in realist fiction in the 1970s which continues into our present day, illustrating children attending government-funded schools as part of their daily life. Particularly in Western countries, these texts address social problems and have become synonymous with social critique or the problem novel (Joosen, 2006). Realistic children’s fiction returned to its didactic roots and was used to make child readers more socially aware, although not all realities were depicted equally; Zipes notes the extra challenges faced by minority populations—including children with disabilities—in the school experience, as these texts predominantly depict white, upper-middle class children (2005, p. 1813).

In examining narratives featuring characters with dyslexia, this study diverges from past research methods on the same subject. Past studies featured a mix of qualitative and quantitative analysis interested in quantifiable representations and identifying the frequency of common traits (Dyches et al., 2001; Prater, 2003; Altieri, 2008; Jozwik and Rice 2020). A common criterion previous studies searched for is the presence of dynamic change or growth in characters with dyslexia (Dyches et al., 2001; Jozwik and Rice 2020). Dynamic characters are considered positive representations, and, compared to other disabilities, dyslexia and other specific learning disabilities are some of the best portrayed (Leininger et al., 2010, p. 590). Although these statistics note that characters with dyslexia are typically dynamic characters, none of these articles analyze how this dynamic growth results from a common narrative pattern.

Therefore, this article employs a narratological perspective focusing on rhetorical choices, cultural narratives, and plot dynamics as opposed to pedagogies or representation. Employing narratology “enables us to distinguish between structural closure (a satisfactory round-up of the plot) and psychological closure, bringing the protagonist’s personal conflicts into balance” (Nikolajeva, 2003, p. 7). As these two aspects commonly coincide in children’s fiction (2003, p. 7), structural and psychological closures coincide within the dyslexic diagnosis paradigm. The use of narrative analysis supports an examination of how these reoccurring narratives influence the dynamic growth of characters with dyslexia while simultaneously reinforcing the power structures which disable these neurodivergent children.

This study examines 16 contemporary realist novels published between 2007 and 2020 featuring at least one adolescent main character with dyslexia, to understand the larger narrative patterns associated with this neurominority (Table 1). These texts are written in English, examining how dyslexia interacts with the Lexism in an anglophone context. Although this corpus was primarily published in the United States of America, some texts were first published in other, primarily anglophone, countries including the United Kingdom, Canada, Australia, and New Zealand. The choice to include a wide an array of anglophone texts was made to understand how dyslexia is perceived within the broader Lexism as opposed to location specific educational systems. From these texts, I have identified one dominant narrative structure: the dyslexic diagnosis paradigm, typified by the prototypical dyslexic diagnosis narrative. Diagnosis is a major theme in many dyslexic narratives and, while previously identified by other scholars (Prater, 2003, p. 56), it has yet to be fully dissected using narrative theory.

**Table 1:** Bibliographic information of contemporary realist children’s and young adult novels featuring a dyslexic character (published 2007–2020)

Title	Author	Year	Country	Publisher	Publishing category
*Close to Famous*	Joan Bauer	2011	USA	Puffin Books	Middle Grade
*End of the Alphabet*	Fleur Beale	2009	New Zealand	Random House New Zealand	Young Adult
*Army Brats*	Daphne Benedis-Grab	2017	USA	Scholastic	Middle Grade
*Waiting for Normal*	Leslie Connor	2008	USA	HarperCollins Children’s Books	Middle Grade
*The Love Letters of Abelard and Lily*	Laura Creedle	2017	USA	Houghton Mifflin Harcourt	Young Adult
*Graffiti Moon*	Cath Crowley	2010	Australia	Pan Macmillan Australia	Young Adult
*Unscripted Joss Byrd*	Lygia Day Peñaflor	2016	USA	Square Fish	Young Adult
*Stupid*	Kim Firmston	2014	Canada	James Lorimer & Company	Hi-Low
*BenBee and the Teacher Griefer*	K.A. Holt	2020	USA	Chronicle Books	Middle Grade
*Fish in a Tree*	Lynda Mullaly Hunt	2015	USA	Nancy Paulsen Books	Middle Grade
*Monday’s Not Coming*	Tiffany D. Jackson	2018	USA	Katherine Tegen Books	Young Adult
*One White Dolphin*	Gill Lewis	2012	UK	OUP Oxford	Middle Grade
*Two-Minute Drill*	Mike Lupica	2007	USA	Puffin Books	Middle Grade
*A List of Cages*	Robin Roe	2017	USA	Hyperion	Young Adult
*Nowhere to Hide*	Kim Sigafus	2019	USA	7th Generation	Hi-Low
*Prove It, Josh*	Jenny Watson	2013	Canada	Sono Nis Press	Middle Grade

## The Prototypical Dyslexic Diagnosis Narrative

The prototypical dyslexic diagnosis narrative contains the pattern in which a character learns they have dyslexia. When distilled into its key narrative elements, the plot follows the three-part structure of struggle, diagnosis, and accommodations—which commonly align with the Aristotelian beginning, middle, and end. Initially, the character struggles to read, although they often portray other areas of competence to illustrate that they are not unintelligent. The character is then diagnosed with dyslexia, uncovering the reason behind their lack of literacy skills. Following their diagnosis, the character is given learning accommodations to better acquire the literacy skills they need to succeed.

Alongside these plot points, we can also trace the character’s emotional character arc as one of shame, declaration, and acceptance. The character is ashamed of their inability to read and these feelings of shame compound until there is a declaration of their frustrations, either by the character or an observant bystander, which often coincides with or leads to their diagnosis. As the character’s dyslexia is addressed, they learn more about their diagnosis and gain literacy skills through learning accommodations until the character reaches a point of acceptance of their dyslexic identity. The twinned progression of these plot and character arcs or structural and psychological closures—struggle/shame, diagnosis/declaration, accommodations/acceptance—demonstrates the prototypical dyslexic diagnosis narrative.

I have analyzed several texts which align with this prototypical diagnosis narrative, including Kim Firmston’s *Stupid* (2016), Lynda Mullaly Hunt’s *Fish in a Tree* (2015), Tiffany D. Jackson’s *Monday’s Not Coming* (2018), and Kim Sigafus’ *Nowhere to Hide* (2019) (Table 2). The following explanation of the prototypical diagnosis narrative will primarily draw examples from *Fish in a Tree* as an exemplar text with supporting examples drawn from the other texts.

**Table 2:** Annotated bibliography of prototypical diagnosis narrative texts

Title	Dyslexic character	Summary
*Fish in a Tree*	Ally Nickerson	Troublemaking Ally disrupts the classroom until a new teacher helps her gain confidence and literacy skills
*Monday’s Not Coming*	Claudia Coleman	When her best friend, Monday, goes missing, Claudia is left to investigate why when no one else seems to notice she’s gone
*Nowhere to Hide*	Autumn	Autumn recognises her dyslexia and prepares her dress and lines for the school play *The Jingle Dress*, with the help of her Aunt Jessie
*Stupid*	Martin	Filmmaker Martin meets parkour enthusiast Stick and together they create a film which they submit to a film contest

### Struggle/Shame

In Altieri’s study of books featuring children with dyslexia, she notes that “[c]haracters often had already experienced failure for a long time or hid their dyslexia from family and professionals for many years” (2008, p. 52). Despite this study examining texts published between 1993 and 2003 (2008, p. 48), Altieri’s statement remains true for this contemporary corpus. Ally experiences both failure within the classroom and attempts to mask her struggles throughout the first half of *Fish in a Tree*. Her poor phonological decoding, typical of people with dyslexia (Grant, 2017, p. 7), is outlined in the first chapter including inconsistent spelling and headaches caused by reading “the brightness of dark letters on white pages for too long” (Hunt, 2015, p. 3). Ally’s poor working memory and processing speeds are illustrated in how she forgets words and needs a substantial amount of time to decode sentences (2015, pp. 13–14). Slow reading speeds are also present in *Monday’s Not Coming* as “[t]he entire class had finished their packet, and [Claudia] was only on page three” (Jackson, 2018, p. 45).

Assuming that she is ‘dumb’ and thus far unaware of her neurodivergence, Ally uses many creative strategies to hide her perceived unintelligence such as turning pages to mimic reading in an ingenious attempt to ‘pass’ as neurotypical to avoid stigmatization (Hunt, 2015, pp. 89–90; Shone, 2020, p. 92). Prater notes how common character strengths, including numeracy, motor, social, and artistic skills, are used to compensate for their literacy deficits, signaling that they have a learning disability and are not unintelligent (2003, pp. 54–55). Ally is good with numbers and a talented artist (Hunt, 2015, pp. 30, 277–296), *Stupid’s* Martin is a talented filmmaker (Firmston, 2016, p. 32), and both *Monday’s* Claudia and *Nowhere*’s Autumn are talented dancers (Jackson, 2018, p.50; Sigafus, 2019, p. 93). While a positive portrayal, Jozwik and Rice warn against this tendency to overly highlight character’s strengths as it positions their neurodivergence as a ‘supercrip’ ability (Schalk 2016). They warn that their disability becomes an acceptable tradeoff when these children have hyper-abilities (Jozwik and Rice, 2020, p. 178).^2^

These talents, however, do not help characters within literacy-heavy environments such as the classroom. Prater comments how misbehaviour is another common trait, especially to cover up deficits in academic performance (2003, p. 55). This observation is supported by Shone who argues that, as schools are a hostile environment for people with dyslexia, they can become the ‘class clown’ as a “maladaptive coping strateg[y]” (2020, p. 91). Ally fits this role, turning her misreading into a ‘purposeful’ prank (Hunt, 2015, p. 22), a tactic also used by *Stupid’s* Martin (Firmston, 2016, p. 71). Bullying is also a common experience (Prater, 2003; Jozwik and Rice, 2020); even without a label, Autumn’s classmates shame her for her academic failure in *Nowhere* (Sigafus, 2019, p. 31).

In addition to their academic struggles, shame exemplifies the character’s feelings of inadequacy. Prater notes how a lack of self-esteem in characters with dyslexia is common (2003, p. 56), an observation Altieri quantified in recording that nearly 70% had low self-esteem, made self-deprecating comments, and had issues pertaining to self-concept (2008, pp. 51–53). The repetition of “*Freak. Dumb. Loser.*” (Hunt, 2015, p. 3, emphasis original) in Ally’s inner monologue echoes throughout *Fish* as she grapples with her own self-image and we see Martin share a similar mantra of “*Loser. Useless. Stupid*.” (Firmston, 2016, p. 171, emphasis original). Shone argues that “before a dyslexic child receives a diagnosis – the child is exposed to the message that they are not worthy of connection and have some unnamed defect which is ‘shameful’” (2020, p. 88) and develop her hypothesized Post-Traumatic School Disorder (PTScD), a variant of Post-Traumatic Stress Disorder (PTSD), which can last into their adulthood (2020, p. 93).^3^ Several texts feature a character’s fear of being placed in alternative academic classrooms: Claudia considers “walking into the Learning Center [as] school suicide” (Jackson, 2018, p. 142); Martin’s father threatens to send him to a military boarding school to “deal with your issues” (Firmston, 2016, p. 130).

### Diagnosis/Declaration

A child with dyslexia is often unaware of the term prior to diagnosis and commonly it is a family friend or acquaintance who suggests a child may have dyslexia rather than another child or even a classroom teacher (Altieri, 2008, p. 49). Altieri observes that teachers are frequently negative figures and portrayed as uncaring or ignorant (2008, pp. 51–52). The teachers in *Stupid* and *Nowhere* fall on the side of ignorance by not considering dyslexia as a cause for either Martin or Autumn’s poor performances (Firmston, 2016, p. 192; Sigafus, 2019, p. 29) or, in the case of Ms. Valette in *Monday*, did not have the time to act upon Claudia’s suspected dyslexia (2018, p. 162). Jozwik and Rice support Altieri’s statement with their updated study of texts published in the 2010s noting how *Fish*’s Mr. Daniels is a rare exception (2020, p. 177). Unlike regular teachers, Mr. Daniels specializes in teaching children with learning differences; his ability to diagnose Ally’s dyslexia based on her literacy habits and classroom behaviour is therefore justified (Hunt, 2015, p. 165). Mr. Daniels peppers his cursory diagnosis with examples of how she’s “a lot smarter than other kids” (2015, p. 157) quoting that: “Everyone is smart in different ways. But if you judge a fish on its ability to climb a tree, it will spend its whole life thinking that it’s stupid” (2015, p. 159). Brown notes how this quote is an allegory of “the impossibility of a completely equal education system” (2019, p. 204) incorrectly attributed to Albert Einstein. Despite problematic aspects, the allusion to this quote in the title and the placement of the quote during this moment of diagnosis emphasizes the narrative importance of this moment.

I do want to highlight that an adult figure is necessary for any formal dyslexia diagnosis, and almost always initiates the testing or at least proposes the possibility of dyslexia to the child (Altieri, 2008, p. 49). Even in atypical circumstances such as *Stupid* where Martin initially takes a dyslexia test online, he only knows to do so because Gloria, his friend Stick’s adult guardian, claims his video is “the best explanation of dyslexia she’s ever seen” (2014, p. 176). Although Martin initially discovers his dyslexic identity through unofficial means, all four texts reference their characters undergoing official testing which happens off-page (Hunt, 2015, p. 160; Firmston, 2016, p. 193; Sigafus, 2019, p. 41; Jackson, 2018, p. 122).

Though diagnosis is an action performed upon the child, the child with dyslexia is not necessarily static. While Ally did not have the language or terminology to describe her struggles prior to Mr. Daniels’ explanation, she still vocalizes her frustrations: “‘But how come I can’t read?’ […] I guess because I am so desperate for an answer” (2015, p. 157). Following Jozwik and Rice’s observation that characters’ desire to read without challenges (2020, p. 174), both Autumn and Martin likewise express their literacy struggles prior to learning about dyslexia. Autumn’s confession is met with empathy from her aunt (Sigafus, 2019, p. 22), however, Martin’s declaration that “words and letters move around on the page” is met with his father’s accusation that he is using drugs (Firmston, 2016, p. 119).

An exception wherein the character does not declare their dyslexia occurs in *Monday*. Claudia’s strong denial leads to the following reaction once her dyslexia is declared by her guidance counselor:

The word [dyslexia] burned through the air—a word that lived on the back of my tongue, gagging me every time I pretended to read a book. A word I had tried to shield and protect myself from for years. But once spoken, it shot out like a hot needle and popped the bubble I lived in. (Jackson, 2018, p. 122)

Whether the word dyslexia is used or not, declaring a character’s literacy struggles creates an ontological shift—the way the character is perceived by their peers and teachers becomes irreversibly changed. This initial declaration is a significant moment, yet the nature of dyslexia as an invisible or hidden disability means these characters continually navigate when and where they should disclose their dyslexia and to whom they own this explanation (Valeras, 2010). While Ally explains her dyslexia to her classmates (Hunt, 2015, p. 182), Claudia fears such admissions, even to her friends (Jackson, 2018, p. 278).

The moment of diagnosis and declaration is brief, but it is the essential turning point for characters with dyslexia. Ally’s entire attitude shifts after her initial diagnosis with Mr. Daniels: “I think about the words ‘learning differences.’ And I’m filled with fear and happiness and questions. But I’m mostly filled with hope” (Hunt, 2015, p. 167). The prototypical diagnosis narrative uses this beat as an epiphanic moment starting the character’s journey to acceptance.

### Accommodations/Acceptance

Altieri rightly states that “no one treatment works for all children with dyslexia” (2008, p. 52). Contemporary texts illustrate characters with dyslexia learning within resource rooms or remedial reading classes, such as Ally’s tutoring sessions with Mr. Daniels or Claudia in the Learning Centre, unlike their twentieth century counterparts which only mentioned these spaces (Prater, 2003, p. 57). There are a multitude of instructional methodologies that can be used to assist people with dyslexia in gaining literacy skills. Prater lists various examples, including: phonics, multisensory instruction, learning strategies, computer programs and tutoring (2003, p. 57). Ally’s phonics education covers syllables and letter formations to teach her how to differentiate similar sounding words such as light, might and night using different coloured letters and incorporates multisensory instruction such as writing with shaving cream (Hunt, 2015, pp. 229–230). The Learning Center provides Claudia with audiobooks and her tutor, Ms. Walker, gives Claudia coloured plastic gel filters to aid with her print reading (Jackson, 2018, pp. 267, 194). A continuing trend Altieri observed is the popularity of out-of-school tutoring (2008, p. 49). Shone advocates for tutoring as it helps rebuild their self-image in addition to gaining literacy skills (2020, p. 96). The private space of tutoring also becomes a healing space, allowing characters like Ally and Claudia to move from an internal place of shame to acceptance of their learning differences.

Jozwik and Rice note that a key “aspect of self-acceptance is the characters’ honest acknowledgement of their reading difficulties and understanding of the steps required for them to manage day-to-day life” (2020, pp. 174–175). Martin’s character arc in *Stupid* exemplifies this when he rejects his father’s idea to send him to a military boarding school. His father’s intentions of “a structured environment with small classes” may be noble, but Martin argues that he would excel at a summer film school as his “dyslexia isn’t only a disability…It means that I see the world differently. It gives me an edge” (Firmston, 2016, pp. 200, 201). With a dyslexia diagnosis, Martin feels more confident in demanding the learning environment and subject which best suits his learning style. While Ally continues to be bullied, she admits that, “I realize that it is easier now that Shay [her bully] and everyone else knows why I have so much trouble” (Hunt, 2015, p. 249). This realization comes after Mr. Daniels discusses famous dyslexic figures as part of their social studies class (2015, pp. 236–243).^4^ Despite the novel’s intention for Mr. Daniels to introduce the concept of dyslexia to Ally’s classmates in a positive light, it inadvertently discloses Ally’s invisible disability without her explicit consent. After the class, several of her classmates approach Ally questioning her further about dyslexia: one considers Ally “lucky” (2015, p. 245) and another “wish[es he] could have it, too” (2015, p. 258). Even if Mr. Daniels' methods are flawed, his lesson does improve Ally’s confidence and further builds her resilience, understanding the necessity of “*Grit*…being willing to fail but try again” and internalizing “how *smart* you have to be to persevere” (2015, pp. 245, 241, emphasis original). Also bullied by their peers, Autumn learns to stand up for herself and memorizes her lines for the school play with minimal effort (Sigafus, 2019, pp. 84–45) and Claudia improves significantly and begins helping her friends at the Learning Centre (Jackson, 2018, p. 427). No matter the accommodations or how many literacy skills gained, every character with dyslexia reaches a point of psychological closure by the novel’s structural conclusion: accepting and better understanding their neurodivergent mind.

The prototypical dyslexic diagnosis narrative illustrates how characters with dyslexia struggle in the traditional classroom, how they are identified and diagnosed, and the accommodations they are given to improve within institutionalized education. The structural and psychological progressions of Ally, Claudia, Martin, and Autumn illustrate a common dynamic arc: starting in a place of shame due to their illiteracy, they declare a desire to learn and end in a place of self-acceptance. This prototype is the simplest iteration of the diagnosis paradigm whereas polytypical dyslexic diagnosis narratives play with these established narrative structures.

## Polytypical Dyslexic Diagnosis Narratives

Before discussing how the diagnosis narrative interplays with the school story genre, I would like to clarify a few common variations of this paradigm. While *Fish* and other prototypical dyslexic diagnosis narratives portray both structural and psychological closures of struggle/shame, diagnosis/declaration, accommodations/acceptance in tandem, there are diagnosis narratives which do not necessarily match this scheme. I have labeled these *poly*typical^5^ dyslexic diagnosis narratives.

A polytypical narrative retains the fundamental psychological development seen in the prototypical narrative; however, it shifts the chronology of the diagnosis. The structural closure of struggle, diagnosis, and accommodations shift temporally outside of the novel’s plot and the point of diagnosis is *not* within the text; the character is already formally diagnosed or has not—and will not be—diagnosed in the text. However, the psychological closure of shame, declaration, and acceptance remains and is essential to the character with dyslexia’s growth.

Two polytypical dyslexic diagnosis narratives I have identified include the pre-diagnosis polytype and the post-diagnosis polytype. The pre-diagnosis polytype features a character who is not formally diagnosed before or within the plot of the novel; however, their character development still tracks the diagnosis paradigm. The post-diagnosis polytype features a character who is diagnosed with dyslexia before the start of the novel, yet their dyslexia is not known to other characters until they declare their learning disability. I will provide a brief example of both polytypes, but it is important to note that while there is naturally room for variance within these narratives, the overarching paradigm of shame/declaration/acceptance remains present.

### Pre-Diagnosis Polytype

The pre-diagnosis polytype occurs when the literacy-challenged character is not officially diagnosed with dyslexia within the plot of the novel, including such texts as Lygia Day Peñaflor’s *Unscripted Joss Byrd* (2016), Fleur Beale’s *End of the Alphabet* (2009), Joan Bauer’s *Close to Famous* (2011) and Cath Crowley’s *Graffiti Moon* (2010) (Table 3). For Joss in *Unscripted*, her lack of diagnosis is a purposeful choice designed by her mother. As an up-and-coming child actress, Joss’ mother rejects the advice that Joss undergo testing as “Joss doesn’t need a label…She needs a career” (Day Peñaflor, 2016, p. 36). The word dyslexia may not even be mentioned in the text, as is the case in *End of the Alphabet* where fourteen-year-old Ruby is only described as having learning problems. Ruby’s shame in her illiteracy defines various aspects of her character both at school and at home. Altieri’s observation of teachers being uncaring or ignorant to various learning disabilities is highlighted when Ruby applies to join the school trip to Brazil (Altieri, 2008, p. 52). While the school principal and guidance counsellor both acknowledge that Ruby has “learning problems,” they still berate her perceived lack of effort on her application essay (Beale, 2009, pp. 78–79). Likewise, neither *Close to Famous* nor *Graffiti Moon* use dyslexia when referring to Foster or Ed’s reading challenges respectively. Both characters experience shame for their inability to read. Foster hides her illiteracy by asking her employer, the aging Hollywood star Miss Charleena, to read for her with the lie that Foster “didn’t bring [her] glasses” (Bauer, 2011, p. 100). Ed completely drops out of school, although his classmates—unaware of his illiteracy—speculate that he was simply uninterested (Crowley, 2011, p. 59).

**Table 3:** Annotated bibliography of pre-diagnosis polytype narrative texts

Title	Dyslexic character	Summary
*End of the Alphabet*	Ruby Yarrow	Underestimated by her family and school, Ruby gets a job and learns Portuguese hoping to earn a spot on her school’s trip to Brazil
*Graffiti Moon*	Ed “Shadow”	Attempting to find her favourite street artist, Shadow, Lucy is accompanied by Ed, a high school dropout who carries many secrets
*Unscripted Joss Byrd*	Joss Byrd	A young up-and-coming actress, Joss struggles with her mother and her undiagnosed dyslexia while attempting to go off script
*Close to Famous*	Foster McFee	Foster and her mother move to the small town of Culpepper where everyone dreams of fame

The climax of two of these novels intersect with a declaration of their learning disability. Layered within Ed’s confession that he is Shadow, the street artist Lucy had been searching for, Ed claims that “Shadow was this great person and I’m nothing … I can barely even read” (Crowley, 2011, p. 257). In *End*, Ruby’s mother publicly acknowledges Ruby’s learning problems as a genetic trait after learning that Ruby’s half-sister on her father’s side is also unable to read at the age of ten (Beale, 2009, pp. 221–222). Ruby’s mother has similarly associated Ruby’s literacy challenges with shame, admitting that she believed her daughter’s illiteracy was “punishment for getting pregnant so young” (2009, p. 222). Positioned at the midpoint of *Unscripted*, Joss confides in her friend Chris that she is “an unofficial dyslexic” (Day Peñaflor, 2016, p. 91). In less of a declaration and more of an agreement, Miss Charleena agrees to teach Foster to read—a task which had “just about split [Miss Charleena’s] brain open”—in exchange for Foster teaching her how to cook (Bauer, 2011, p.109).

For pre-diagnosis polytypes, it is common for narratives to not illustrate learning accommodations—although Joss creates audio recordings of her scripts to memorize (Day Peñaflor, 2016, p. 113)—and these novels end with the characters’ acceptance of their own strengths and weaknesses. For *End*’s Ruby, she prepares for a solo trip to Brazil after learning to speak Portuguese and is comforted that her family now understands that her lack of literacy skills does not correlate to her intelligence. In both *Close* and *Graffiti*, each character ends with a plan to improve their literacy skills: Foster is prepared to meet her new school’s specialized reading teacher (Bauer, 2011, p. 226), and Ed plans to attend a continuing education program at a local college where he hopes to learn with the help of a scribe (Crowely, 2011, p. 298). Despite how pre-diagnosis polytypical narratives may or may not explicitly mention dyslexia or learning disabilities, characters such as Joss, Ruby, Foster, and Ed still follow the dyslexic diagnosis paradigm in how the characters understand and accept their neurodivergent mind.

### Post-diagnosis Polytype

The post-diagnosis polytype is where the character with dyslexia is officially diagnosed before the plot of the novel begins, in such texts as Jenny Watson’s *Prove It, Josh* (2013), Leslie Connor’s *Waiting for Normal* (2008), and Mike Lupica’s *Two-Minute Drill* (2007) (Table 4). However, while the character is aware of their dyslexia and how it affects their literacy abilities, their peers are not. In this polytype, the character with dyslexia chooses to mask their learning disability for a variety of reasons and therefore follows the same psychological closure of shame, declaration, and acceptance of the diagnosis paradigm again within the plot of the novel.

**Table 4:** Annotated bibliography of post-diagnosis polytype narrative texts

Title	Dyslexic character	Summary
*Prove It, Josh*	Josh Parker	Josh makes a bet that he will beat his classmate in an upcoming boat race or he will have to read aloud at the library’s Literacy Day
*Two-Minute Drill*	Chris Conlan	Unlikely friends, the brainy, clumsy Scott and star quarterback Chris, team up to help each other succeed in both football and school
*Waiting for Normal*	Addie Schmeeter	Addie and her mother, who has bipolar disorder, live in a small trailer in the city, separated from Addie’s beloved stepfather and half-sisters

The titular character of *Prove It, Josh* chooses to hide his dyslexia from his new classmates when he moves to the west coast of Canada, hoping that a different learning environment with smaller class sizes and fewer distractions would be helpful (Watson, 2013, p. 149). Despite already receiving accommodations, including private tutoring, Josh is still ashamed of his dyslexia and his classmates single him out as a “*Dumb. Stupid. Idiot.*” (2013, p. 22, emphasis original). In *Waiting*, Addie’s shame is manifested within the concept that she does not share her mother’s ‘Love of Learning’. More equated to a love of reading, this phrase becomes a stand-in for Addie’s perceived unintelligence as she struggles through vocabulary books and learns to play the flute by ear, rather than reading sheet music (Connor, 2008, pp. 22, 26).

In *Two-Minute*, the character with dyslexia, Chris, is not the focal protagonist and the reader is therefore not initially privy to his feelings of shame. The reader, alongside protagonist Scott, learns of Chris’ dyslexia when he declares he may have to quit football season “[b]ecause [he] can’t read” (Lupica, 2007, p. 59). The declarations in *Prove It* come from both supporting characters and the protagonist. Mrs. Gordon, the grandmother of his sailing partner, Dakota, recognizes Josh’s dyslexia as she had helped Dakota’s father as a child with his dyslexia and offers to tutor Josh (Watson, 2013, pp. 88–90). Josh’s later declaration to Dakota is not met with stigma, noting that her father also has dyslexia (2013, p. 125). While Josh never meets Dakota’s father, he becomes a figure who helps Josh accept his own dyslexia, knowing that others have accepted it before him.

Addie’s initial declaration is atypical as she does not mention dyslexia directly, referring to her learning challenges as “*spatial relationship* problems” (Connor, 2008, p. 70, emphasis original). She reflects that this declaration is “a relief” because she is telling her supportive adult friends from the neighbouring minimart rather than her classmates (2008, p. 70). Oddly, Addie is only told she has dyslexia much later in the text when discussing her problems at school with her stepfather (2008, p. 212). This conversation coincides with her stepfather affirming that Addie does have the ‘Love of Learning,’ but that it manifests differently from her mother (2008, p. 213).

After receiving extra tutoring from Mrs. Gordon and Dakota, Josh embraces his dyslexic identity, confident enough in his own ability to read aloud at the library’s literacy day (Watson, 2013, p. 152). With help from Scott, Chris improves his grades and feels more confident after his reading test (Lupica, 2007, p. 97). Despite Josh, Addie, and Chris’s prior diagnoses, their novels follow the psychological closure of the diagnosis paradigm.

## Considering the School Story

Now that this diagnosis narrative and its various polytypes have been identified, it is essential to understand that the genre of the school story *encourages* this paradigm. Reimer makes an astute observation about school stories:

Criticism of schools as places of injustice, unhappiness, and coercion have featured in narratives from the beginning of the genre, but such critiques have been a comparatively thin threat through the tradition. More typical is the story in which the new scholar learns first to understand, then to accept, and finally to excel at, the ways of the strange world he or she is entering. (2009, p. 224)

The pattern Reimer identifies as ‘learn, accept, excel’ is perhaps a broader identification of ‘struggle, diagnosis, accommodations’. As the school story prepares youth to become citizens, the dyslexic diagnosis narrative also pushes its neurodivergent youth along the same path. Reimer rightly critiques the school system and its history as a capitalist machine that functioned “proudly and explicitly…[as] ‘the chief nurseries’ for the [British] empire” (2009, p. 215) throughout the nineteenth century, traditions of which continue today.

Disability inherently defies the central tenants of capitalism—how all citizens must work and produce to have value—and in that sense neurodiversity has perhaps emerged as a political label to fight against twenty-first century neoliberalism (McGee, 2012, p. 13). Scholar Mitzi Waltz investigates the historical pressures placed on children coming of age within capitalism. Waltz states that the ‘normal’ child has only existed for about a hundred years due to the convergence of compulsory schooling and mass conscription of young men in World War I (2020). Underpinning both developments is the Industrial Revolution and capitalist boom-bust cycles which required a child who would not become a social burden or criminal, and which therefore defined normalcy in terms of medicine, social work, education, and parenting. The child becomes a product as “modern industry (like modern warfare) demanded adults who could be slotted into industrial processes as a standardised component” (2020, p. 18). Under this paradigm, the system of diagnosis is inevitably classist. When considering dyslexia, Waltz notes how there are often dubious diagnoses in well-off families to help their children succeed in university exams as opposed to poor families where the diagnosis promotes “B-track life” or the school-to-prison pipeline (2020, p. 24). To further add capitalist insult to injury, “[e]ven when normalcy cannot be produced by the system, its failures can be monetised as students and clients” (2020, p. 24) through expensive testing, lengthy private tutoring, and pricey technological accommodations. Skipping forward to the present day, Waltz notes that “[w]hile labels can theoretically be used to provide support that allows each child to flourish, the desire for standardised outcomes tends to guide the form and goals of support in education systems as they currently exist” (2020, p. 22). Diagnosis enables disruptive students like *Fish*’s Ally to be reintegrated into the normal classroom. While supplemental accommodations are provided to her, these are done without straining the systems already in place. Ally and other characters with dyslexia learn to better fit the system rather than the system better adapting to suit their learning needs.

Zipes ends his introduction to the school story with a reflection on reality:

A look at today’s schools in British and North America can tell us quite a bit about the societies that support them (or fail to do so), as they change because of the demands of and new conditions in a fast-paced, diverse, and ever-changing world. (2005, p. 1816)

As Zipes also discusses how school stories of the twentieth and twenty-first century began to show cracks in how schools—and by extension home life—fail to support children (2005, p. 1813), these changes are perhaps more driven by the desires of neoliberalism rather than desires of the student body. Despite their framing as a success story wherein the troubled character with dyslexia learns to read, these narratives only cement the status quo of current educational systems, rather than try to find alternative means of education to better suit all minds and abilities. The system acknowledges that individual students are failing, yet they are still intelligent enough to have potential if identified and given appropriate accommodations to remain suitably competitive within their peer group. These characters end their novels as more productive workers, no longer disrupting the school setting, and therefore may proceed along the school-to-workforce pipeline.

While all the characters with dyslexia saw improvement, readers finish the texts with an understanding that these characters will *continue* to struggle throughout their education even with any accommodations they may be granted. They must accept not just their dyslexia, but how their dyslexia will remain an obstacle to their ability to learn within their educational system. As Shone comments, “a metacognitive understanding of the processing issues related to dyslexia and how these issues are understood and interpreted in society is vital if the dyslexic person wants to transform the way they operate in the world” (2020, p. 96). This places the onus on the individual child to be responsible for their own learning and perceived deficit—how they must mold themselves with the aid of accommodations to fit the status quo—enforcing the medical model of disability at an institutional level.

Hunt’s *Fish in a Tree* comments upon how the systems of literacy today are still steeped in history when Ally’s class discusses Noah Webster during a field trip to a colonial schoolroom. Noah Webster is described to the class as a “visionary” for creating the first American spellers and dictionaries, to which Ally responds: “Some visionary. This spelling stuff is all *his* fault, since he’s the one who got it in his head that we all needed to spell the same way” (Hunt, 2015, p. 153, emphasis original). However brief, this passage illustrates Hunt’s acknowledgement of how standardized education developed in the Industrial Revolution, as Waltz described, remains today, and has left dyslexic students like Ally behind. Mr. Daniels may introduce alternative teaching methods into his classroom but, ultimately, he is still trapped in the same institution—one teacher cannot change Lexism.

## The Dyslexic Persistence Narrative

Despite some of these diagnosis narratives illustrating many of the flaws within current educational practice—such as the rage felt by Claudia’s parents for their daughter’s dyslexia “fl[ying] under the radar” (Jackson, 2018, p. 123) or Martin’s initial mis-diagnosis of ADHD for which the doctor prescribed Ritalin, “a little pill to make all my problems go away” (Firmston, 2016, p. 75)—these instances are often sparse and ultimately do not alter the diagnosis paradigm. True nuance to this diagnosis paradigm becomes more present after the diagnosis narrative has occurred; narratives not defined by the same structural and psychological closures but by how they interrogate literacy norms within the school systems these characters with dyslexia must continue attending. In Collinson and Penketh’s study interviewing postgraduate students with dyslexia, they observe that a recurrent narrative which enables students with dyslexia to continue with formal education is that of “resistance through persistence” (2010, p. 15). They suggest that “rather than being described as stories of triumph over adversity, […] the need for an ongoing engagement in forms of resistance appears to be a necessary form of action” (2010, p. 14). The diagnosis paradigm emphasizes this triumph over adversity narrative: the diagnosis leads to improvement both in the character’s reading abilities and self-esteem. There are certainly elements of persistence in the diagnosis narratives—Jozwik and Rice note that it is a common, and positively viewed, trait of characters with dyslexia (2020, p. 173)—but, while these narratives end with an underlying understanding that children with dyslexia will have to continue to muster this resilience indefinitely, there remains an emphasis on the triumph of learning to read. The dyslexic diagnosis paradigm positions the medical model’s ability to solve the ‘problem’ of dyslexia as a triumph. The process of diagnosis promoted in these narratives emphasizes initial improvement rather than continual struggle; after all, a diagnosis means the dyslexic character can be ‘treated’ accordingly.

Building from Collinson and Penketh’s phrase, I have identified another less common paradigm found within the school story genre: the dyslexic persistence narrative. Persistence narratives start after the diagnosis narrative has concluded. The dyslexic character has undergone the cycle of struggle/shame, diagnosis/declaration, accommodations/acceptance but still finds themselves struggling throughout their education. While elements of shame^6^ and disclosing can be present within these texts, themes of acceptance/accommodations are generally at the forefront to an extent wherein the plot remains distinct from the post-diagnosis polytype. Persistence narratives have the potential to act as what Mitchell and Snyder term “*disability counternarratives*” (2014, p. 164, emphasis original). Counternarratives critique the “inherently ‘flawed’ narrative of ‘cure or kill’ formulas” witnessed in normalcy narratives (2014, p. 165). They combine Leonard Davis’ argument that disability within narratives signals an abnormality in need of correction with Frank Kermode’s ‘sense of an ending’—which postulates that all narratives seek a resolution—to understand how narratives have historically sought to ‘fix’ disabled characters as “the end of the abnormal is always located in its restitution within the fold of the normal” (2014, p. 164). While there is no ‘cure’ for dyslexia, the sense of ending from diagnosis narratives promote the idea that the character’s dyslexia must be ‘fixed’ enough so that they are no longer too abnormal for the system, rendering them normalcy narratives. As persistence narratives counter this false happy ending that the diagnosis paradigm portrays, they can function as counternarratives illustrating that there is no ‘quick fix’ to systemic inequity in literacy education.

It is important to note that not all persistence narratives function as counternarratives. There are some persistence narratives which feature character(s) with dyslexia, yet their dyslexic identity often does not play a large role in their characterization or the narrative. For example, Tom from Daphne Bendis-Grab’s *Army Brats* (2017) has dyslexia, yet this aspect of his identity is not mentioned past page 60 of the text. If texts like *Army Brats* normalize the dyslexic experience, counternarratives interrogate the normalcy of Lexism itself. One common trait many persistence narratives share which aligns them with counternarratives is the presence of multiple neurodivergent characters featured within the story-world. This can be multiple characters with dyslexia or, more commonly, the addition of more neurominorities or other disabilities wherein characters form a disabled/neurodiverse community (Table 5). With the greater presence of disabled characters, there are naturally more opportunities to discuss and question the disabled experience as opposed to defaulting these explanations onto able-bodied and neurotypical people. These conversations allow for a shift away from the individual to a community—from an individual, medical problem to a societal issue.

**Table 5:** Annotated bibliography of persistence narrative texts

Title	Dyslexic character	Secondary character(s) with disabilities	Summary
*Army Brats*	Tom Bailey	None	The Bailey family move to an army base in Virginia. Siblings Tom, Charlotte, and Rosie investigate the mysteries of Fort Patrick
*BenBee and the Teacher Griefer*	Ben B. (Benjamin Bellows) / Ben Y. (Benita Ybarra)	Jordan “J” Jackson (ADHD), Javier Julio Jimenez (disfluency)	Four sixth-grade students form a tight friendship when reluctantly placed in summer school together with their unorthodox teacher, Ms. J
*A List of Cages*	Julian Harlow	Adam Blake (ADHD)	Reuniting with his estranged foster brother in high school, Adam rescues Julian from an abusive situation
*The Love Letters of Abelard and Lily*	Lily Michaels-Ryan	Abelard Mitchell (autism), Lily (co-occurrence of dyslexia, ADHD, and dysgraphia)	Lily and Abelard, two neurodivergent high schoolers, meet in detention and discover their mutual interest in love letters and each other
*One White Dolphin*	Kara Wood	Felix Andersen (cerebral palsy)	Kara and her new friend Felix work with veterinarians and specialists to help save a baby albino dolphin caught in old fishing netting to reunite her with her mother

Although another full essay is needed to do justice to these persistence narratives, an evocative example as to how they nuance the diagnosis paradigm is present within K.A. Holt’s *BenBee and the Teacher Griefer* (2020). The situation of Ben B, the titular character and one of four protagonists, illustrates the *failure* of diagnosis and the medical model. When Ben B begins summer school, he has no official diagnosis attached to his incoming report card. Dysgraphia is mentioned, but there is also a note reading “why no 504?” (Holt, 2020, p. 2).^7^ Ben had been tested for dyslexia years prior, yet the test came back “*Inconclusive*” and “no one had any answers.” (2020, pp. 208, 207). Without an official diagnosis, he was not eligible for learning accommodations despite an obvious need.

*BenBee*’s example illustrates how diagnosis is an imperfect system excluding as many students as it helps. As Chapman notes: “on the one hand, we do need to be able to distinguish between minority forms of functioning and genuine pathology; but on the other hand, any attempt at definition risks being harmful or exclusionary” (2020, p. 219). Excluding Ben B from a standardized definition such as dyslexia,^8^ AD(H)D, or other learning disability becomes a medical technicality further preventing him from succeeding. Ben B’s narrative epitomizes the counternarrative as he *fails* to be diagnosed, exposing flaws within the current system of diagnosis.

Alongside exposing this major flaw within the diagnosis system, *BenBee* demonstrates how the social model of disability can interject these school stories. Ms. J utilizes ‘positive niche classroom construction’, a term Armstrong borrowed from biology which advocates that neurodiverse students can and should act upon their learning environments to create favourable conditions to acquire literacy skills: choosing reading materials that interest them and practicing sentence formation and spelling in a gamified, co-operative setting (2012, p. 13). The concept of niche construction within a classroom setting acts as a counternarrative as it demonstrates alternative means of schooling. As opposed to forcing students into traditional classroom settings, niche construction creates settings where students are set up to learn rather than fail, with the bonus of promoting student agency. Again, while another full essay is needed to fully explore how *BenBee* and other persistence narratives use the social model of disability to resist traditional and disabling methods of literacy instruction, Ms. J’s resistance is ultimately unsustainable and nearly leads to her termination (Holt, 2020, p. 247). Like *Fish*’s Mr. Daniels, one teacher cannot dismantle the educational system to save Ben B from failing the Florida State Test yet again and Ms. J’s efforts are for nought.

## Conclusion

Zipes states that, “[t]heories about education have always informed the school story” (2005, p. 1806). When discussing educational theory surrounding dyslexia, Altieri further notes that her “study indicates that this issue [the need to diagnose and assess students with dyslexia] may be as prevalent in literature as it in society” (2008, p. 52). Current institutional education systems still use the deficit model of disability requiring failure before one can receive support (Shone, 2020, p. 92). These ableist environments set up people with dyslexia to fail before they can be assessed and reintegrated into the educational system. Even persistence narratives which strive to interrogate and propose alternatives to current disabling forms of literacy education inevitably bow to the mimetic nature of the realist genre: to portray a truly inclusive space in an institutional education setting would not read as true.

The dyslexic diagnosis paradigm reflects and reinforces this institution. The reaffirming endings through the acceptance of their neurodivergence may create dynamic characters with dyslexia, but these narratives support and normalize a flawed system. The school story thus reinforces the status quo, telling the reader “the ways of the strange world” (Reimer, 2009, p. 224), rather than encouraging readers to question *why* this world must be so strange. Yet, can the school story accomplish such a utopic goal? If the proverb on “judg[ing] a fish by its ability to climb a tree” (Brown, 2019, p. 204) is to be believed, a completely equal education system is impossible. The inequalities will remain as will the dyslexic diagnosis paradigm. It remains a school story, not a student story.

## Footnotes

1 While not all the texts within this corpus are wholly set within the school, all these texts are haunted by institutional education. For example, Joan Bauer’s *Close to Famous* (2011) is set during the summer months, but Foster is terrified of returning to school (2011, p. 82).

2 Jozwik and Rice specifically refer to “unnatural athletic talents” (2020, p. 178). An example of this from my corpus includes Chris’ quarterback prowess in *Two-Minute Drill* (2007) referenced in the post-diagnosis polytype section.

3 While Post-Traumatic Stress Disorder (PTSD) is recognized by DSM-5, Post-Traumatic School Disorder (PTScD) is *not* currently recognized as a diagnosis or condition by psychological, educational, or legal professions at the time of writing. PTScD is a working hypothesis by Shone who has built her argument upon Scott’s previous studies which observed that children with dyslexia could experience both acute and complex PTSD concurrently due to their schooling environments (2004).

4 *Stupid* also references many famous dyslexics, although Firmston only references actors and athletes (2016, p. 202).

5 Following the term’s usage in biological taxonomy, a polytypism indicates the existence of several types or subspecies within a species (OED, 2006). I have repurposed this word to describe the variances of this diagnosis narrative.

6 Shone’s “mechanism of shame” (2020, p. 85) describes how shame and trauma continue past the point of diagnosis for many people with dyslexia.

7 This is in reference to a Section 504 plan. According to the Florida Department of Education, a 504 plan describes the accommodations that a school must legally provide to support the education of a student with disabilities (2011).

8 Whereas both dyspraxia and ADHD have a consensus definition, Grant notes that there is no such agreement for the criteria of dyslexia. Because of the similarities in reading challenges, Grant warns that we “need to avoid automatically assuming that all reading difficulties are due to dyslexia” (2017, p. 28).
